# A roadmap of apical bud formation in white spruce identifies potential regulators of time to bud set

**DOI:** 10.1186/1753-6561-5-S7-O21

**Published:** 2011-09-13

**Authors:** Janice Cooke, Walid El Kayal, Betty Pelgas, Irina L Zaharia, Suzanne Abrams, Nathalie Isabel

**Affiliations:** 1 University of Alberta, Canada; 2 Natural Resources Canada, Canadian Forest Service, Laurentian Forestry Centre, Canada; 3 Plant Biotechnology Institute, National Research Council of Canada

## Background

Bud development is an adaptation that temperate forest trees have acquired to survive inclement winter conditions and resume growth the following spring. Bud development is a complex physiological and developmental process comprising bud formation, cold and desiccation tolerance development, and dormancy acquisition [1]. Because bud formation is accompanied by growth cessation, the timing of bud formation represents a trade-off between acquisition of winter hardiness and duration of active growth [2]. This is particularly true for determinate species such as white spruce (*Picea glauca* [Moench] Voss), where preformed stem units contained within the apical bud constitute most of next season’s growth. Timing of bud set in white spruce shows genetic variation [3]. Our goal is to identify genes that exert genetic control over time to bud set in white spruce. As first steps towards that goal, we have conducted microarray gene expression profiling of shoot tips during the transition from active growth to dormancy in white spruce, and identified candidate regulators of bud formation by comparing these differentially expressed (DE) genes to results obtained from QTL analyses of time to bud set conducted using the same genes as molecular markers.

## Materials and methods

Two year old white spruce nearing completion of active growth were placed in either short day (SD; 8 h days / 16 h nights, 20°C) or continued long day (LD; 16 h days / 8 h nights, 20°C) photoperiods, and samples harvested at 0, 1, 3, 7, 14, 28 and 70 d. After an additional 8-15 weeks in SD, plants were transferred to low temperatures (LT) under continued SD for 3-4 weeks prior to final harvest. Light microscopy, microarrays, SYBR Green qRT-PCR, and hormone analyses were conducted as described [4].

## Results and discussion

Unlike indeterminate species such as *Populus* spp., white spruce does not require environmental cues such as reduced photoperiod or cooler temperatures to initiate bud formation, although these cues may be required to complete bud development and acquire endodormancy. However, SD hastens and synchronizes bud formation. To reduce biological variation, we conducted most analyses with seedlings under SD. Microarray analyses revealed 4460 DE genes in shoot tips during SD-induced bud formation. These analyses revealed the timing of molecular processes that occur during bud formation, identifying DE groups of genes associated with meristem transitions, identity and maintenance; organogenesis; protein synthesis and turnover; chromatin assembly and remodeling; cell wall biosynthesis and expansion; carbohydrate and lipid metabolism; secondary metabolism; and general stress responses. Changes in gene expression correlated with anatomical changes occurring at the shoot apex during the transition from active growth to endodormancy. Comparison of DE genes in developing buds under SD and LD uncovered ca. 2000 genes that are DE under both photoperiods at comparable phases of bud formation. This common set of genes is likely important for bud formation, particularly during early stages. Many genes DE shortly after transfer of plants to SD are likely photoperiod-responsive but not essential for bud formation. Genes DE only under SD at later stages of bud formation could be involved in processes associated with completion of bud formation and transition to endodormancy, events which do not appear to occur fully under LD. Further comparison of shoot tip, needle and stem transcript abundance profiles during this time course revealed approximately 100 genes that are DE only in developing buds and show greater transcript abundance in developing buds than other tissues. Several genes DE during bud formation – including some genes both preferentially expressed and only DE in buds – are putatively associated with hormone signalling. Quantification of ABA, cytokinins, auxin and their metabolites during SD-induced bud formation revealed distinct profiles for these hormones during bud formation, with shifts in hormone levels coinciding with morphological events such as bud scale development. A small number of genes DE in developing buds fall within QTL regions identified for time of bud set [5]. Some of these genes also show signatures of selection in natural populations of white spruce via identification of Fst outliers [6]. These candidate genes may exert genetic control over time to bud formation, and are currently being studied in more detail An eQTL experiment and association study are underway to identify additional candidate regulators of time to bud set.

## Conclusions

This comprehensive study of gene expression changes that occur at the shoot apex during the transition from active growth to dormancy in white spruce has revealed timing of processes important for overwintering preparation, and groups of genes implicated in these processes. Use of these same genes as molecular markers in QTL analyses has enabled us to tentatively identify candidate genes that may exert genetic control over the time of bud formation in white spruce. These results provide new insight into an important adaptive trait, and lay the groundwork for more detailed studies into regulators of time of bud set in conifers.
